# Comparative retrieval analysis of antioxidant polyethylene: bonding of vitamin-E does not reduce in-vivo surface damage

**DOI:** 10.1186/s12891-021-04898-y

**Published:** 2021-11-30

**Authors:** Dominic T. Mathis, Joshua Schmidli, Michael T. Hirschmann, Felix Amsler, Johann Henckel, Harry Hothi, Alister Hart

**Affiliations:** 1 Department of Clinical Research, University of Basel, 4051 Basel, Switzerland; 2 Department of Orthopaedic Surgery and Traumatology, Kantonsspital Baselland (Bruderholz, Liestal, Laufen), 4101 Bruderholz, Switzerland; 3 Amsler Consulting, 4059 Basel, Switzerland; 4 Institute of Orthopaedics and Musculoskeletal Science, University College London, Royal National Orthopaedic Hospital, Stanmore, UK

**Keywords:** Total knee arthroplasty, Polyethylene, Retrieval analysis, Polyethylene surface damage, Surface roughness

## Abstract

**Background:**

With the Persona® knee system a new polyethylene formulation incorporating vitamin-E which aims to reduce oxidation and maintain wear resistance was introduced. Although in-vitro studies have demonstrated positive effects of the vitamin-E antioxidants on UHMWPE, no retrieval study has looked at polyethylene damage of this system yet. It was the aim to investigate the in-vivo performance of this new design, by comparing it with its predecessor in retrieval analysis.

**Methods:**

15 NexGen® and 8 Persona® fixed-bearing implants from the same manufacturer (Zimmer Biomet) were retrieved from two knee revision centres. For retrieval analysis, a macroscopic analysis of polyethylene using a peer-reviewed damage grading method was used (Hood-score). The roughness of all articulating metal components was measured using a contact profilometer. The reason(s) for TKA revision were recorded. Statistical analyses (t-test) were performed to investigate differences between the two designs.

**Results:**

The mean Hood score for Persona® inserts was 109.3 and for NexGen® 115.1 without significant differences between the two designs. Results from the profilometer revealed that Persona® and NexGen® femoral implants showed an identical mean surface roughness of 0.14 μm. The Persona® tibial tray showed a significantly smoother surface (0.06 μm) compared to the NexGen® (0.2 μm; *p* < 0.001). Both Hood score and surface roughness were influenced by the reasons for revision (*p* < 0.01).

**Conclusions:**

The bonding of the antioxidant vitamin-E to the PE chain used in the novel Persona® knee system does not reduce in-vivo surface damage compared to highly crosslinked PE without supplemented vitamin-E used in its predecessor knee system NexGen®. However, the Persona® titanium alloy tibial tray showed a significantly smoother surface in comparison to the NexGen® titanium alloy tibial tray. This study provides first retrieval findings of a novel TKA design and may help to understand how the new Persona® anatomic knee system performs in vivo.

## Background

A novel total knee system, introduced to the European market in 2012, was developed to improve the mechanics of the knee replacement by making a more anatomically accurate knee implant [1, 2]. A new highly crosslinked ultrahigh-molecular-weight polyethylene (UHMWPE) formulation incorporating the antioxidant vitamin-E (alpha-tocopherol) aims to reduce oxidation and maintain wear resistance and strength throughout the life of the implant [3].

In-vitro studies have demonstrated positive effects of vitamin-E stabilized PE in different total knee arthroplasty (TKA) designs [4–6]. Furthermore, various kinematic and biomechanical studies have demonstrated the design benefits of the novel anatomic knee system [7–10]. In addition, data from registries and clinical studies show excellent survivorship and clinical outcomes at 1 to 3 years [1, 11]. The predecessor to the novel anatomic knee design represents a successful and proven implant for TKA with scientific evidence for its remarkable clinical long-term outcome [12]. It uses inserts of type highly crosslinked UHMWPE [13]. Comparative studies between these two knee systems are very scarce in literature [1, 14]. To date there is no comparative retrieval analysis between the components of the novel anatomic knee system and its predecessor.

To justify the use of the novel knee system over its well-established predecessor, post-market surveillance by means of retrieval data are vital as emphasized by the new Medical Device Regulation [15]. Therefore, the primary aim of this retrieval study was to assess the magnitude of damage of the vitamin-E stabilized PE used in the novel anatomic knee system in comparison to highly crosslinked UHMWPE used in the predecessor knee system. Secondly, the surface roughness of the tibial and femoral component was analysed and compared. It was hypothesized that there would be no significant difference between the two designs in terms of PE damage and implant surface roughness.

## Methods

### Retrieval cohort

This study examined all Persona® (*n* = 8) and NexGen® (*n* = 15) TKA implants consecutively received at our centre since 2016; all are produced by a single manufacturer (Zimmer Biomet, Warsaw, Indiana, USA). The implants were removed either in a specialized knee revision centre in Finland or in Switzerland by one fellowship trained knee surgeon in each centre. All implants received at the retrieval centre were included in the study with the exception of implants revised due to infection (limited comparability).

TKA specifications and patient demographics for each case are presented in Table 1.

**Table 1:** Patient demographics

Case number	Gender	Age, yrs	Time to revision, yrs	Reason(s) for revision	Design, Type	Revision surgeon
1	F	51.3	0.4	Patellofemoral, stiffness, malalignment	NexGen®, PS	Surgeon 1
2	M	50.9	3.0	Instability	Persona®, PS	Surgeon 1
3	F	71.1	1.0	Instability, patellofemoral	Persona®, CR	Surgeon 1
4	F	76.6	9.0	Instability	NexGen®, PS	Surgeon 2
5	F	61.8	5.9	Periprosthetic fracture	NexGen®, CR	Surgeon 2
6	F	71.3	5.9	Instability	NexGen®, CR	Surgeon 2
7	F	67.3	7.8	Malalignment	NexGen®, CR	Surgeon 2
8	M	70.6	1.6	Instability	Persona®, PS	Surgeon 1
9	F	67.8	3.5	Instability, stiffness	Persona®, CR	Surgeon 1
10	F	69.2	1.4	Instability, patellofemoral	Persona®, PS	Surgeon 1
11	F	66.3	14.8	Progression of OA	NexGen®, PS	Surgeon 2
12	F	72.2	1.5	Instability, patellofemoral	Persona®, PS	Surgeon 1
13	F	52.4	0.9	Stiffness	NexGen®, CR	Surgeon 2
14	F	69.3	1.9	Instability	NexGen®, PS	Surgeon 2
15	F	79.6	9.7	Instability	NexGen®, CR	Surgeon 2
16	M	69.3	5.8	Instability	NexGen®, CR	Surgeon 2
17	F	84.2	10.1	Instability	NexGen®, CR	Surgeon 2
18	F	79.8	14.1	Instability	NexGen®, CR	Surgeon 2
19	F	72.8	13.1	Instability	NexGen®, CR	Surgeon 2
20	F	80.2	1.2	Stiffness	NexGen®, CR	Surgeon 2
21	M	70.5	3.2	Instability, malalignment	Persona®, CR	Surgeon 1
22	F	58.3	18.1	Instability	NexGen®, PS	Surgeon 2
23	F	66.0	2.2	Instability	Persona®, CR	Surgeon 1

*SD* standard deviation, *OA* osteoarthritis, *PS* posterior stabilized, *CR* cruciate retaining

The novel anatomic implants (Persona®) consisted of two different designs (cruciate-retaining (CR, *n* = 4) and posterior-stabilized (PS, *n* = 4)); the femoral component made from cobalt chromium (CoCr), the tibial tray from titanium alloy (Ti-6Al-4 V). All eight patients had a fixed bearing (FB). All tibial inserts were made of vitamin-E highly cross-linked polyethylene with antioxidant protection (Vivacit-E®, Zimmer Biomet, Warsaw, Indiana, USA). These implants were retrieved from five (62.5%) female and three (37.5%) male patients, with a mean (standard deviation, SD) age of 67.3 (± 6.9) years and a mean (SD) time to revision of 2.2 (± 0.9) years. The main reason for revision was instability (*n* = 8, 100%; Table 2).

**Table 2:** Patient demographics by implant type

Design, type	Gender (F:M)	Age at revision, mean and SD (yrs)	Time to revision, mean and SD (yrs)	Reason(s) for revision
Persona®, total	5:3	67.3 (± 6.9)	2.2 (± 0.94)	Instability (*n* = 8, 100%); patellofemoral problem (*n* = 3, 37.5%); malalignment (*n* = 1, 12.5%); stiffness (*n* = 1, 12.5%)
Persona®, CR	3:1	68.9 (± 2.4)	2.5 (± 1.1)	Instability (*n* = 4, 100%); patellofemoral problem (*n* = 1, 25%); malalignment (*n* = 1, 25%); stiffness (*n* = 1, 25%)
Persona®, PS	2:2	65.7 (± 10)	1.9 (0.7)	Instability (*n* = 4, 100%); patellofemoral problem (*n* = 2, 50%)
NexGen®, total	14:1	69.4 (± 10.1)	7.9 (± 5.5)	Instability (n = 9, 60.0%); stiffness (*n* = 3, 20%); malalignment (*n* = 2, 13.3%); others (periprosthetic fracture, progression OA, *n* = 2, 13.3%); patellofemoral problem (*n* = 1, 6.6%)
NexGen®, CR	9:1	71.9 (± 9.7)	7.4 (± 4.4)	Instability (*n* = 6, 60%); stiffness (*n* = 2, 20%); malalignment (*n* = 1, 10%); others (periprosthetic fracture *n* = 1, 10%)
NexGen®, PS	5:0	64.4 (± 9.8)	8.9 (± 7.7)	Instability (*n* = 3, 60%); stiffness (*n* = 1, 20%); malalignment (*n* = 1, 20%); others (progression OA *n* = 1, 20%); patellofemoral problem (*n* = 1, 20%)

*SD* standard deviation, *OA* osteoarthritis, *PS* posterior stabilized, *CR* cruciate retaining, the percentages totalled > 100% because some knees had more than one reason for revision recorded

The predecessor implants (NexGen®) consisted of two different designs: CR-Flex (*n* = 10), and Legacy® posterior-stabilized (LPS)-Flex (*n* = 5), all with a FB design, the femoral component also made from CoCr, the tibial tray from titanium alloy (Ti-6Al-4 V). The tibial inserts were all made of Prolong® highly crosslinked UHMWPE (Zimmer Biomet, Warsaw, Indiana, USA). These implants were retrieved from 14 (93%) female and one (7%) male patients, with a mean (SD) age of 69.4 (±10.1) years. The main reason for revision was also instability (*n* = 9, 60%) and the mean (SD) time to revision was 7.9 (±5.5) years. The latter was significantly longer for the predecessor compared to the novel anatomic knee system (*p* < 0.01; Table 2).

Institutional and ethical approval was obtained and patients gave informed consent for participation in the study (2019–02031). The study performed in accordance with the ethical standards of the responsible committee and with the guidelines of the Helsinki Declaration of 1975, as revised in 2008.

### Sample preparation

All components were decontaminated using 10% formaldehyde solution (Solmedia Ltd., UK), followed by rinsing with water. The tibial tray backside and stem surfaces were prepared by using methylated spirit 99% (Solmedia Ltd., UK) to gently remove biomaterial without affecting cement adhesion.

### Study design

**Fig. 1:**
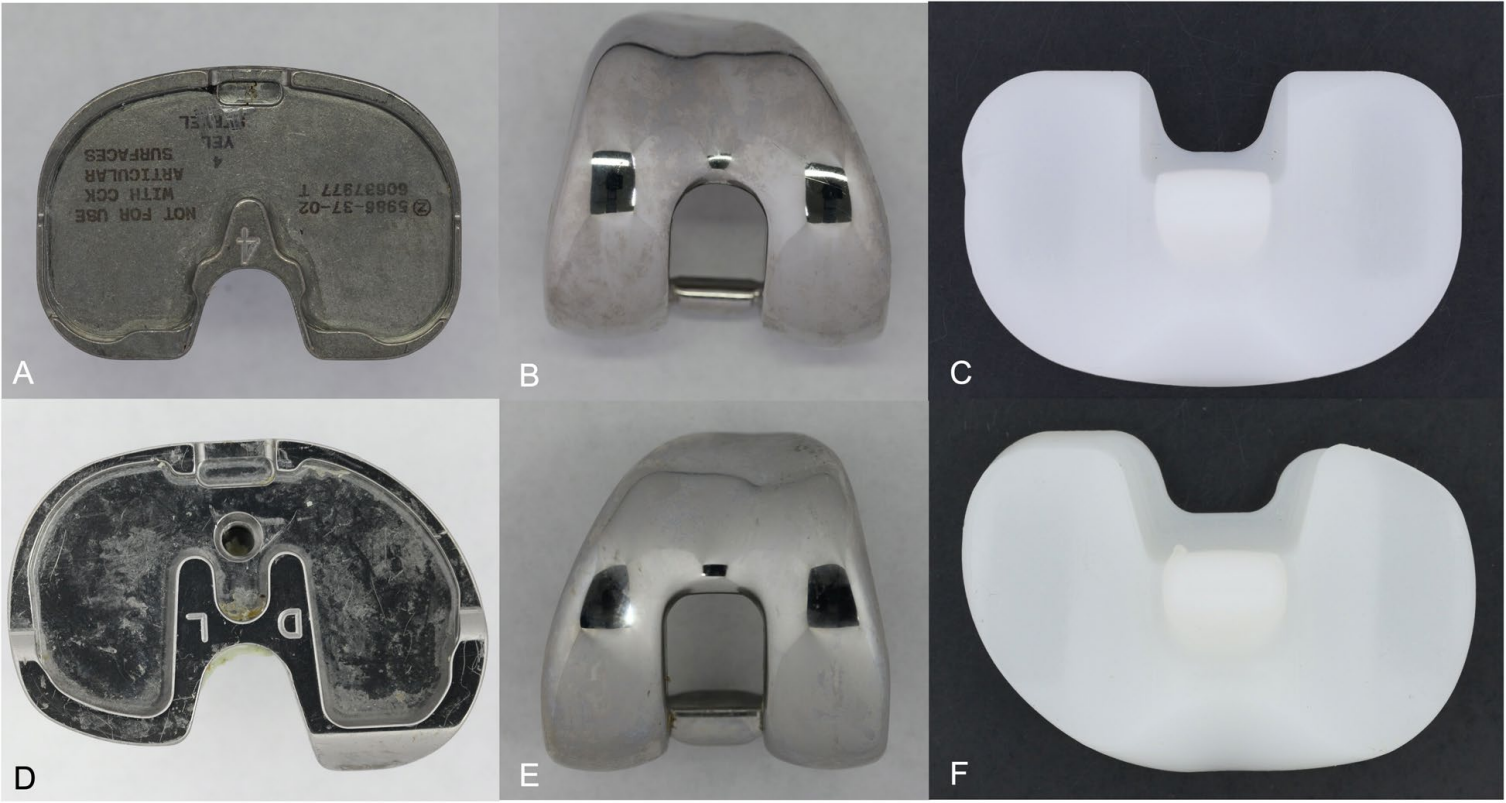
Examples of the two knee implant designs involved in the study: **A**-**C** predecessor knee system NexGen®; **D**-**F** novel anatomic knee system Persona® **A** CoCr fixed bearing tibial tray, **B** CoCr femoral shield, **C** polyethylene tibial insert posterior-stabilized, **D** CoCr fixed bearing tibial tray, **E** CoCr femoral shield, **F** polyethylene tibial insert posterior-stabilized

**Fig. 2:**
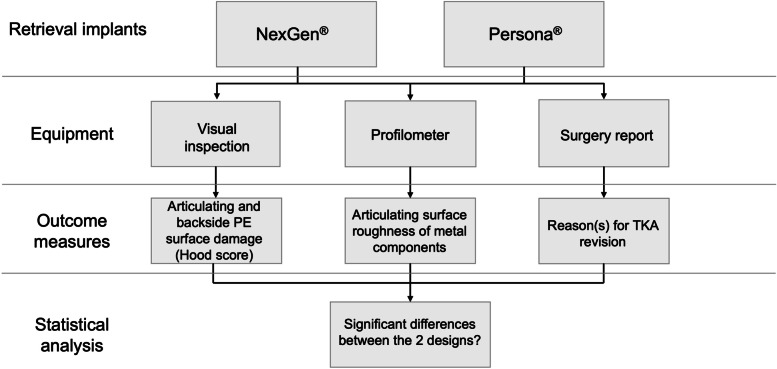
Flow chart showing the study design; PE, polyethylene; TKA, total knee arthroplasty

The following retrieval analyses were performed: (1) macroscopic analysis of PE components, using a peer-reviewed damage grading method, (2) measured roughness of metal components and (3) compared findings between the two knee designs (Fig. 1). In addition, reason(s) for TKA revision were collected from surgery reports (Fig. 2).

### Surface damage in polyethylene tibial inserts (Hood score)

**Fig. 3:**
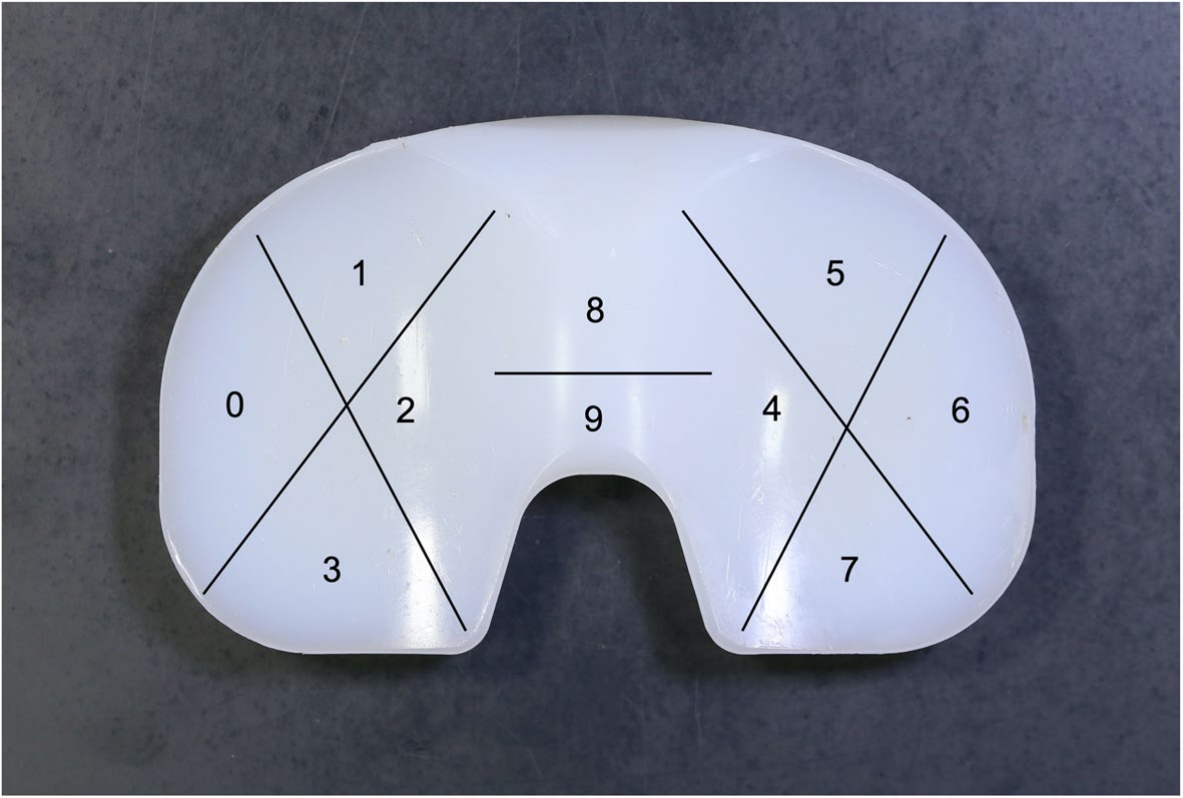
Surface division according to the Hood score

All the polyethylene tibial inserts were visually investigated and the surface damage on both articulating and backside surfaces was assessed using the Hood Score [16]. This grading system consists of dividing both the articulating and backside surfaces into 10 sections and grading each of them according to the presence and severity of seven modes of surface damage (surface deformation, pitting, embedded debris, scratching, burnishing, abrasion and delamination). The surface division is shown in Fig. 3. The maximum damage grade possible is 21 for a single section (grade 3 for each of the seven damage modes) and 210 for the entire surface (grade 3 for each of the seven damage modes for each of the 10 sections).

Articulating and backside surface scores were assessed, as well as the overall score as sum of the previous two. For statistical interpretation section 0–3, 4–7 and 8–9 were grouped as medial, lateral and central. Mean values for each design were calculated (Table 3).

**Table 3:** Mean values and standard deviations (SD) of surface roughness values and hood scores of all implants investigated; N of all revision reasons

Implant type	Total (*N* = 23)	NexGen® (*N* = 15)	Persona® (*N* = 8)	Comparison		Comparison – corrected for time to revision	
Surface roughness (Ra) and Hood Score	Mean, SD	Mean, SD	Mean, SD	R^2^	P	R^2^	P
Tibial lateral Ra	0.14 (+/−0.07)	0.18 (+/−0.04)	0.07 (+/−0.04)	0.68	.000	0.65	.000
Tibial medial Ra	0.15 (+/− 0.08)	0.2 (+/− 0.05)	0.06 (+/− 0.01)	0.77	.000	0.71	.000
Tibial lateral & medial Ra	0.15 (+/−0.07)	0.19 (+/− 0.04)	0.06 (+/− 0.03)	0.78	.000	0.75	.000
Femoral lateral Ra	0.14 (+/− 0.03)	0.15 (+/− 0.04)	0.13 (+/− 0.01)	0.09	.166	0.20	.046
Femoral medial Ra	0.14 (+/− 0.03)	0.14 (+/− 0.03)	0.14 (+/− 0.03)	0.01	.745	0.06	.291
Femoral lateral & medial Ra	0.14 (+/− 0.02)	0.14 (+/− 0.03)	0.14 (+/− 0.02)	0.02	.484	0.20	.046
Hood Score articulating lateral	22.2 (+/−4.8)	22.1 (+/−4.7)	22.7 (+/−5.7)	0.00	.805	0.04	.387
Hood Score articulating central	12.8 (+/−3.1)	13.2 (+/−3.0)	11.8 (+/−3.7)	0.04	.381	0.00	.833
Hood Score articulating medial	23.9 (+/−7.2)	23.6 (+/−8.2)	24.7 (+/−4.6)	0.00	.769	0.01	.617
Hood Score articulating total	59 (+/−10.2)	58.9 (+/−9.4)	59.2 (+/−11.0)	0.00	.953	0.03	.475
Hood Score backside lateral	22.4 (+/−4.5)	22.9 (+/− 4.6)	21.3 (+/− 4.4)	0.03	.490	0.04	.410
Hood Score backside central	10.9 (+/−3)	11.0 (+/−3.3)	10.5 (+/−2.2)	0.01	.740	0.02	.590
Hood Score backside medial	21.2 (+/−5.7)	22.3 (+/−5.8)	18.3 (+/−4.7)	0.11	.149	0.14	.105
Hood Score backside total	54.5 (+/−10.9)	56.2 (+/−11.3)	50.2 (+/−9.2)	0.07	.261	0.10	.183
Hood Score lateral total	44.7 (+/−7.2)	44.9 (+/−6.3)	44.0 (+/−9.7)	0.00	.795	0.00	.956
Hood Score medial total	45.1 (+/−10.5)	45.9 (+/−11.9)	43.0 (+/− 6.3)	0.02	.577	0.01	.616
Hood Score overall total	113.4 (+/−17.1)	115.1 (+/−17.1)	109.3 (+/−18.0)	0.02	.502	0.01	.674
Reason for revision	N (%)	N (%)	N (%)	R^2^	P	R^2^	P
Instability	17 (73.9)	9 (60.0)	8 (100)	0.19	.039	0.29	.014
Malalignment	3 (13.0)	2 (13.0)	1 (12.5)	0.00	.957	0.00	.795
Patellofemoral	4 (17.4)	1 (7.0)	3 (37.5)	0.15	.068	0.03	.463
Stiffness	4 (17.4)	3 (20.0)	1 (12.5)	0.01	.669	0.11	.153
Others (periprosthetic fracture, progression OA)	2 (8.7)	2 (13)	0 (0)	0.05	.301	0.01	.689

Comparison between the two implant designs using R^2^. *P*-values < 0.05 were considered statistically significant

### Articulating surface roughness of metal components (profilometer)

**Fig. 4:**
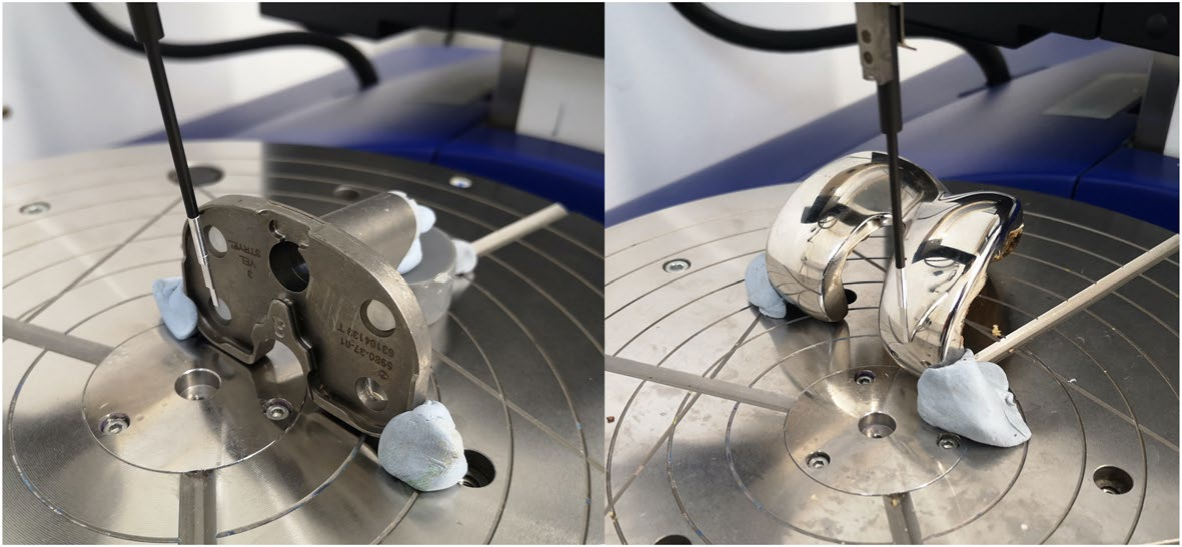
Example of surface roughness analysis performed using a contact profilometer

To measure the articulating surface roughness of metal components, a contact profilometer Talyrond 365 (Taylor Hobson, Leicester, UK) with a 5-μm probe was used. Surface roughness is defined as the average of the absolute values of the surface height deviations measured from the mean plane. Each metal component was positioned on the spindle and six vertical traces (length = 10 mm; number of points = 10,000) were acquired on the articulating surface, avoiding areas damaged by scratches created during the revision surgery (Fig. 4). Mean values for each design were calculated (Table 3).

### Statistics

Results are presented as means, ranges and standard deviations (SD) for interval data and with numbers and percentages for binary variables. All statistical tests were two-tailed and *p*-values < 0.05 were considered statistically significant. To compare mean values, t-tests for independent samples for group differences were used, e.g. comparison of the two component types, and t-tests for paired samples for comparison of different values within the same subject, e.g. medial vs. lateral or articulating vs. backside. Pearson correlations were calculated for interval data, phi coefficients to compare binary variables. Results are also presented as scatter- and box-plots.

Due to the possible influence of time to revision on outcome variables, the differences between implant types were additionally corrected for time to revision for all parameters investigated using partialized values.

A post hoc analysis using G*Power, version 3.1.9 (University of Kiel, Germany) tested for correlations, that, for the given *N* = 23, an effect size rho =0.53 can be found with a power of 80% with a two-sided p of 0.05.

All data were analysed by an independent professional statistician using SPSS Statistics for Windows, version 26.0 (Armonk, NY: IBM Corp, USA).

## Results

**Fig. 5:**
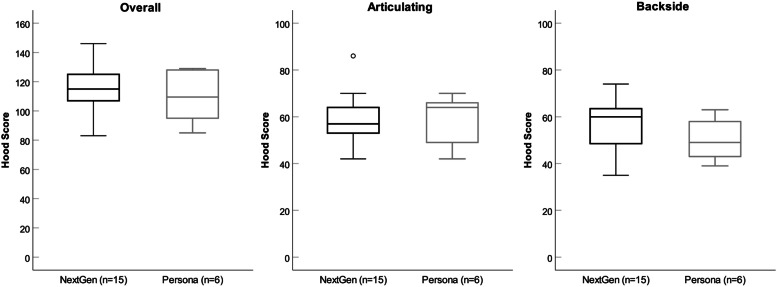
Graphs showing the comparison of overall, articulating and backside surface Hood score between NexGen® and Persona® tibial inserts (fixed bearings)

The mean overall Hood score (SD) of the tibial inserts for the entire cohort was 113.4 (±17.1), for Persona® 109.3 (±18.0) and for NexGen® inserts 115.1 (±17.1). The mean (SD) values for articular and backside surfaces were 59.0 (±10.2) and 54.5 (±10.9), respectively. None of the Hood scores differed significantly between implant types, even when the data were corrected for time to revision (Table 3). The majority of the tibial inserts showed the same Hood score (± 1 SD) on the medial and lateral side (*n* = 9, 43%) as well as on the articular and backside surface (*n* = 11, 52%). 33% (*n* = 7) showed higher Hood scores on the lateral, 24% (*n* = 5) on the medial side, whilst 38% (*n* = 8) had higher Hood scores on the articular and 10% (*n* = 2) on the backside surface. With regard to Hood score, there were no significant differences between medial and lateral (*p* = 0.88), articular and backside surfaces (*p* = 0.11) or between the two designs (*p* = 0.60; Table 3, Fig. 5).

Results from the contact profilometer revealed that Persona® and NexGen® *femoral* implants showed both an identical mean (SD) surface roughness value of 0.14 μm (± 0.02; Table 3). No significant differences in medial and lateral femoral surface roughness were found between the implants either (*p* = 0.48). Seventy-four percent (*n* = 17) showed the same roughness values (± 1 SD) on the medial and lateral femoral surface, only in 13% (*n* = 3) the lateral roughness value was higher than the medial one and in 13% (n = 3) vice versa.

**Fig. 6:**
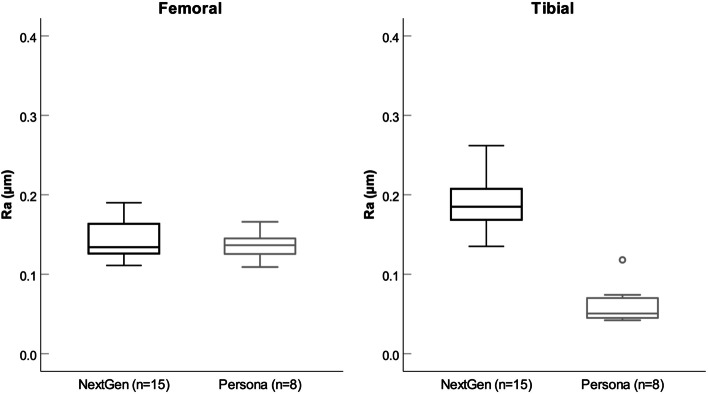
Graphs showing the comparison of articulating surface roughness (Ra) of femoral and tibial components between NexGen® and Persona® implants

Conversely, Persona® titanium FB *tibial* trays showed a significantly smoother surface (medial 0.06 ± 0.01 μm, lateral 0.07 ± 0.04 μm and overall 0.06 ± 0.03 μm) compared to the NexGen® titanium FB tibial trays with overall 0.19 ± 0.04 μm (medial 0.2 ± 0.05 μm, lateral 0.18 ± 0.04 μm; *p* < 0.001; Table 3, Fig. 6). Ninety-one percent (*n* = 21/23) of the analysed tibial tray surfaces showed the same medial and lateral roughness values (± 1 SD). With regards to surface roughness of the tibial trays, there was no significant difference between the medial and lateral articulating surface (*p* = 0.63).

Correlations between surface roughness, Hood score, implant type, reason for revision and time to revision are demonstrated in Table 4. The most important ones include the following: Time to revision was significantly longer in NexGen® implants (*p* < 0.01) and shorter when the reason for revision was a patellofemoral problem (*p* < 0.05). Apart from that, there is no evidence, that the time to revision has a significant influence on the surface roughness or Hood score of the implant. In the Persona® group, instability was described as a reason for revision significantly more often (p < 0.05) than in the NexGen® group. Implants revised due to instability showed significantly lower surface roughness values of the tibial articulating tray (*p* < 0.01), whereas the tibial inserts of implants revised because of malalignment or patellofemoral problems showed significantly lower Hood scores (*p* < 0.01). There was no significant correlation between Hood score and surface roughness. Neither was a significant correlation found for the Hood score between the front- and backside of the tibial insert nor between the roughness of tibial and femoral implant surfaces.

**Table 4:** Pearson correlation between implant design, time to revision, reason for revision, surface roughness and Hood score. *** *p* < 0.001, ** *p* < 0.01, * *p* < 0.05

Pearson correlation		Implant type (Persona® - NexGen®)	Time to revision	Reason for revision				Roughness		Hood score		
				Instability	Malalignment	Patellofemoral	Stiffness	Tibial total	Femoral total	Articulating total	Backside	Overall total
Implant type (Persona® - NexGen®)		1***	−0.53**	0.43*	−0.01	0.39	−0.09	−0.88***	−0.15	0.01	−0.26	−0.16
Time to revision		−0.53**	1	0.09	− 0.16	− 0.43*	− 0.4	0.34	− 0.39	0.26	− 0.03	0.14
Reason for revision	Instability	0.43**	0.09	1	−0.36	0.01	−0.51*	− 0.53**	− 0.25	0.2	0.23	0.26
	Malalignment	−0.01	−0.16	− 0.36	1	0.16	0.16	−0.04	0.01	−0.22	−0.62**	− 0.52*
	Patellofemoral	0.39	−0.43*	0.01	0.16	1	0.09	−0.31	0.13	−0.6**	−0.43	− 0.63**
	Stiffness	−0.09	−0.4	− 0.51*	0.16	0.09	1	0.18	0.52*	−0.06	0.14	0.05
Roughness	Tibial total	−0.88***	0.34	−0.53**	−0.04	− 0.31	0.18	1	0.06	−0.11	0.26	0.1
	Femoral total	−0.15	−0.39	− 0.25	0.01	0.13	0.52*	−0.06	1	−0.07	0.32	0.16
Hood score	Articulating total	0.01	0.26	0.2	−0.22	−0.6**	−0.06	− 0.11	−0.07	1	0.32	0.8***
	Backside	−0.26	−0.03	0.23	−0.62**	− 0.43	0.14	0.26	0.32	0.32	1	0.82***
	Overall total	−0.16	0.14	0.26	−0.52*	−0.63**	0.05	0.1	0.16	0.8***	0.82***	1

The cross-prosthesis analysis of effects between PS and CR types did not show any significant differences in any of the investigated parameters.

## Discussion

This retrieval study is the first to examine components of a novel anatomic knee system and compare them with retrieval findings from its predecessor. Our most important finding was that no significant difference of the surface damage between Persona® vitamin-E stabilized and NexGen® highly crosslinked UHMWPE inserts was found; however, significantly higher surface roughness values were found for the articulating tray of NexGen® in comparison to Persona®. The findings of this study do not suggest any association between surface roughness of the metal components and the tibial PE inserts nor a dependency of the measured parameters on time to revision. Rather, the magnitude of the implant damage is associated with the reasons for the TKA revision.

The highly crosslinked PE of Persona® is actively stabilized with the anti-oxidant vitamin-E to prevent oxidative degeneration of the PE and maintain wear resistance and strength throughout the life of the implant [3]. Different studies conducting accelerate ageing and knee simulator tests reported the superior performance of anti-oxidant doped PE in terms of wear resistance, oxidation resistance and stability of material properties when compared with crosslinked ultrahigh-molecular-weight polyethylene (UHMWPE) [4–6, 17]. Testing showed ultra-low wear with a 73% wear reduction of Vivacit-E® PE compared to re-melted highly crosslinked PE [18]. Conversely, in a previous in-vitro study Micheli et al. reported that after 5 million cycles both vitamin-E doped and crosslinked UHMWPE tibial inserts without supplemented vitamin-E, with fixed bearing designs, showed similar evidence of scratching and burnishing on both condylar and backside surfaces [17]. In a more recent study, Cerquiglini et al. confirmed these findings, highlighting that UHMWPE tibial inserts (fixed bearings) showed no significant differences in the overall and articulating surface damage compared to anti-oxidant doped PE [19]. However, all the anti-oxidant tibial inserts included in Cerquiglini study were made of AOX™ polyethylene (DePuy Synthes, Warsaw, IN, USA), incorporating the COVERNOX™ anti-oxidant (PBHP, pentaerythritol tetrakis [3-(3,5-di-tert-butyl-4-hydroxyphenyl)propionate]), whereas Persona® inserts analysed in this study were stabilized with the antioxidant vitamin-E (alpha-tocopherol).

Our findings are in line with these studies: there was no significant difference between the Hood score of all measured tibial PE surfaces of NexGen® and Persona®. Whereas Cerquiglini et al. reported that the majority of the tibial inserts showed higher Hood scores on the medial side, the present study found the same scores on all surfaces analysed. In this regard, on the basis of our data, it could be speculated that due to the significantly shorter time to revision in the Persona® group, hood score and roughness values would have been higher after a longer period. However, this is pure speculation, further studies with longer revision intervals are needed to test this hypothesis. Furthermore, in this context, the reason for the revision can be discussed. In both this study and the Cerquiglini study, instability was the main reason for revision. In literature instability is associated with increased PE wear, in particular asymmetric extension instability in the varus aligned TKA resulting in accelerated medial PE wear [20]. Thus, these results point out the wear resistance and stability of the PEs used in this cohort with symmetrical damages despite instability problems.

Results from the contact profilometer revealed that there was no significant difference in the medio-lateral deformation of the femoral CoCr components between NexGen® and Persona®. However, the significant difference found in the surface roughness of the tibial trays with regards to a smoother surface in Persona® titanium FB tibial trays seems to be linked to design difference instead of being material-related: both tibial tray designs are made of the same material titanium alloy and the PEs backside damages did not show differ significantly between the two designs. By contrast, the locking mechanism in the tibial FB tray used in Persona® has four locking areas (“Quadra-Lock”), whereas the NexGen® system only has three (“Tri-Lock”). Therefore, it can be speculated that the “Quadra-Lock” mechanism of Persona® has smaller differences in room of movement between PE and tibial tray allowing micromotion and potential damages of the tray. Furthermore, these differences of tibial tray surface roughness might be attributed to the most obvious design alteration with the anatomical tibial tray in the Persona® design as opposed to the symmetrical tray in NexGen®.

However, our results must be contrasted with the findings of Lapaj et al., who found higher backside damage scores of inserts retrieved from NexGen® FB TKAs compared to the control group (Stryker Triathlon, Smith&Nephew Genesis II, Stryker Scorpio, DePuy PFC Sigma, Aesculap Search Evolution) [21]. These findings have been supported by the results of Cerquiglini et al. in 2019 showing significantly higher Hood scores on the backside surface of Attune® tibial FB inserts compared to their PFC® counterparts (DePuy Synthes, Warsaw, IN, USA) [19]. Both authors attributed the higher backside damages scores to changes in design features.

The present study also analysed correlations between the parameters investigated. Interestingly, time to revision did not show any significant correlations with Hood scores or surface roughness values. Rather than the damage and roughness of the implant, the reason for revision seems to affect the time to revision. Patients revised due to patellofemoral problems showed a significantly shorter time to revision compared to patients with other pathologies, such as instability or malalignment. This finding is in accordance with the National joint registry which lists patella problems first for early revisions of primary TKA [22]. As compared to NexGen®, Persona® is much shorter on the market, it is rather an intuitive finding that there is a correlation between NexGen® and time to revision. Persona® implants were significantly more revised due to instability problems, NexGen® implants did not reveal any associated reasons for revision. It is also noticeable that surgeon 1 stated several reasons for revisions in most patients, while surgeon 2 gave only one reason at a time. Thus, the surgeon and his clinical setting represents a possible bias that may explain differences in the respective indications for TKA revision [23]. However, it is pure speculation whether the reason for revision depends on the surgeon of the primary TKA, the surgical technique, the patient or the prosthesis itself.

The comparison of Hood score of the tibial insert and the surface roughness of all metal implants revealed no significant correlations. This is in agreement with previous results of Scholes et al. who analysed the surface roughness and Hood score of 19 cobalt-chromium-molybdenum-on-UHMWPE prostheses [24]. Neither they found a correlation between Hood score and surface roughness nor with time to revision.

Our study has limitations, similar to all retrieval studies. First, our sample size was small; however, this was the first study of its kind for the Persona® knee system and these data can be used for sample size calculations in future studies. Second, the Hood score is a semi-quantitative score used to assess surface damage, which was recently proved to be only a moderate predictor of material loss [25]. The main drawback of the Hood score is that it does not provide any information regarding PE wear, which is expressed as either gravimetric or volumetric loss of material [26]. Third, we focused only on measurements strictly related to the articulating part of the knee joint, without investigating other factors such as the backside of the metal implants, cement adhesions and dimensions of the implants. These features will be content of future studies. Fourth, the evaluation of the clinical benefit remains completely outside the scope of this study. Long-term clinical and longer-term retrieval studies will be necessary to elucidate any clinical advantages of using Persona® knee implants including vitamin-E doped tibial inserts. Finally, due to the limited availability of retrieval data of Persona® implants, a certain heterogeneity in the cohort could not be avoided (different reasons for revision and times to revision).

## Conclusions

These first-time comparative retrieval findings of two knee systems from the same manufacturer suggested that the bonding of the natural antioxidant vitamin-E to the PE chain used in the Persona® knee system does not reduce in-vivo surface damage compared to highly crosslinked PE without supplemented vitamin-E used in its predecessor knee system NexGen®. However, the Persona® titanium alloy tibial tray showed a significantly smoother surface in comparison to the NexGen® titanium alloy tibial tray.

These retrieval findings provide novel insights into a new TKA design recently introduced to the market. However, future analysis is required to examine the implant in a larger sample and more multidimensional.

## Data Availability

No data is presented other than in this article.

## Abbreviations

UHMWPE: Ultrahigh-molecular-weight polyethylene
TKA: Total knee arthroplasty
PE: Polyethylene
PS: Posterior stabilized
CR: Cruciate retaining
MB: Mobile bearing
FB: Fixed bearing
SD: Standard deviation
CoCr: Cobalt chromium
